# An experimental study on the flocculating settling of unclassified tailings

**DOI:** 10.1371/journal.pone.0204230

**Published:** 2018-09-25

**Authors:** Jiwei Bian, Hao Wang, Chongchun Xiao, Deming Zhang

**Affiliations:** 1 School of Resources and Safety Engineering, Central South University, Changsha, People's Republic of China; 2 Department of Civil Engineering, University of Ottawa, Ottawa, ON, Canada; 3 Feny Corporation Limited, Changsha, People's Republic of China; Stockton University, UNITED STATES

## Abstract

Unclassified tailings are the main backfilling aggregates in mines and their settling is the first step in the utilization of tailings; thus, it is very important to determine their settling behavior. The aim of this study was to understand the flocculating settling behavior of unclassified tailings with different factors. The combination of property detection, laboratory experiments and industrial tests were used to assess the flocculating settling behavior of unclassified tailings; the orthogonal experimental design and the control variate method were used for an experimental design. The results show that the flocculating settling velocity of unclassified tailings decreases with the increase of slurry concentration and that this settling velocity increases first and then decreases with the increase of flocculant unit consumption. The underflow concentration is positively correlated with the slurry concentration and negatively correlated with the flocculant unit consumption and flocculant concentration. Slower feed velocity could produce higher concentration underflow but lower clarity overflow water. The greater the mud height, the higher the underflow concentration and the suspended solids concentration in the overflow water. The underflow concentration has a maximum at the rake speed of 0.3 r/min, and the rake speed has little effect on the suspended solids concentration in the overflow water. By analyzing the settling velocity, the underflow concentration, the suspended solids concentration in the overflow water and the solid flux, the following parameters of the flocculating settling experiments were determined: the flocculant type is APAM with a molecular weight of 12 million, the flocculant unit consumption is 30 g/t, the slurry concentration is 6 vol.%, the flocculant concentration is 0.1 wt.%, the rake speed is 0.3 r/min, and the feed velocity is 0.4 L/min (its solid flux is 0.523 t/(m^2^·h)). The industrial tests were carried out based on the laboratory settling data, and the appropriate selection parameters of the industrial tests were estimated.

## Introduction

Mine backfilling plays a significant role in improving ground stability, increasing ore recovery, and controlling underground pressure and thus has both economic and environmental benefits [1–3]. Therefore, mine backfilling is being widely used all over the world in many underground mines [4–6]. The development of backfilling technology and equipment has provided strong support for the popularization and application of mine backfilling. The key technology of mine backfilling is the preparation of backfilling slurries (the mixture of backfilling aggregate, cementitious materials and water [7, 8]). Tailings are the mine wastes that are discharged after the ore is milled to select the valuable concentrate [9]. The tailings are used as backfilling aggregate to fill underground voids, which not only can solve the source problem of backfilling aggregate in many mines but also save many land resources and reduce the environmental hazards of tailings reservoir and surface yard [10–12]. Generally, tailings backfilling, especially cemented backfilling, requires the mass concentration of backfilling slurry reach more than 65%. However, the concentration of tailings discharged from beneficiation plant is generally only 10%~25%, which cannot directly be used to prepare high-concentration backfilling slurries. The settling and thickening of tailings have become one of the main research directions for mine backfilling.

Traditionally, the settling and thickening of tailings rely on gravity settling in the vertical sand silo. A number of advanced technologies are used to increase the extraction rate of minerals by grinding the crushed ore into increasingly fine particles [13]. More than 80% of tailings particles have size less than 74 μm in some mines. Relying on only gravity settling, the settling velocity is very slow, and the underflow concentration is low; thus, gravity settling is unable to meet the requirements of the continuous supply of tailings and preparation of high-concentration backfilling slurries. Moreover, the suspended solids concentration in the overflow water is high, which is unable to meet the environmental requirements. It is convenient and feasible to increase the settling velocity of tailings by adding flocculants; therefore, flocculating settling technology has been widely used in the area of tailings settling [14, 15].

In recent years, many scholars have conducted a large number of research works on the flocculating settling of tailings. Henderson and Wheatley [16] demonstrated that the intrinsic viscosity (indirectly, the molecular weight) has a significant effect on the settling velocity of flocculated tailings with anionic polyacrylamides. Jiao et al. [17] studied the effects of feed concentration and the flocculant unit consumption on the settling velocity and the settling concentration through the static flocculating settling test and constructed a simple settling velocity model. Franks et al. [18] conducted continuous solid-liquid separations with the temperature responsive flocculants poly, which produced higher concentration underflow but lower clarity overflow and significantly reduced underflow rheology. Eswaraiah et al. [19] studied the settling characteristics of iron ore slurry with anionic, cationic, and nonionic flocculants at different ranges of slurry pH values and found that the flocculants increase the settling rate by several times and the anionic flocculants is more effective in enhancing the slurry settling rate. Selomuya et al. [20] used optical confocal scanning laser microscopy and high-resolution X-ray microtomography methods to probe 3D visualizations of the microstructure of flocculated particulates and sediments. Wang et al. [21] introduced magnetic treatment into crude tailings slurry settling and found that appropriate magnetization conditions could enhance the settling indices of crude tailings slurry. Bürgera et al. [22] studied continuous thickening of flocculated suspensions in vessels with varying cross-section, including divergent or convergent conical vessels, and a numerical algorithm was employed for simulations of continuous thickening. Many works have been performed in the field of settling and thickening of tailings. However, it is inaccurate to evaluate settling behaviors on basis of the settling velocity as the only evaluation index in most studies, and there are few research studies on the settling behavior of tailings under continuous feeding and continuous discharging.

The flocculating settling is a complex physical and chemical process [16], influenced by many factors, such as particle size, feed concentration, flocculant type, flocculant unit consumption, slurry chemistry and slurry concentration. The main objectives of this study are: (1) to determine flocculant types that are suitable for the tailings through flocculant selection experiments; (2) to analyze the effects of slurry concentration, flocculant unit consumption and flocculant concentration on the flocculating settling of unclassified tailings through the static cylinder experiments; (3) to analyze the effects of feed velocity, mud height and rake speed on the flocculating settling of unclassified tailings through the dynamic experiments on the basis of static cylinder experiments; (4) to decide the flocculating settling parameters of engineering applications.

## Experimental programs

The slurry concentration mentioned in the paper is the volume concentration, and the underflow concentration is the mass concentration.

### Materials and mixture proportioning

#### Materials

(1) Tailings

The tailings utilized in this study were a type of copper tailings, which were received from Yinshan Copper Mine, located in Jiangxi Province, China.

**Fig 1:**
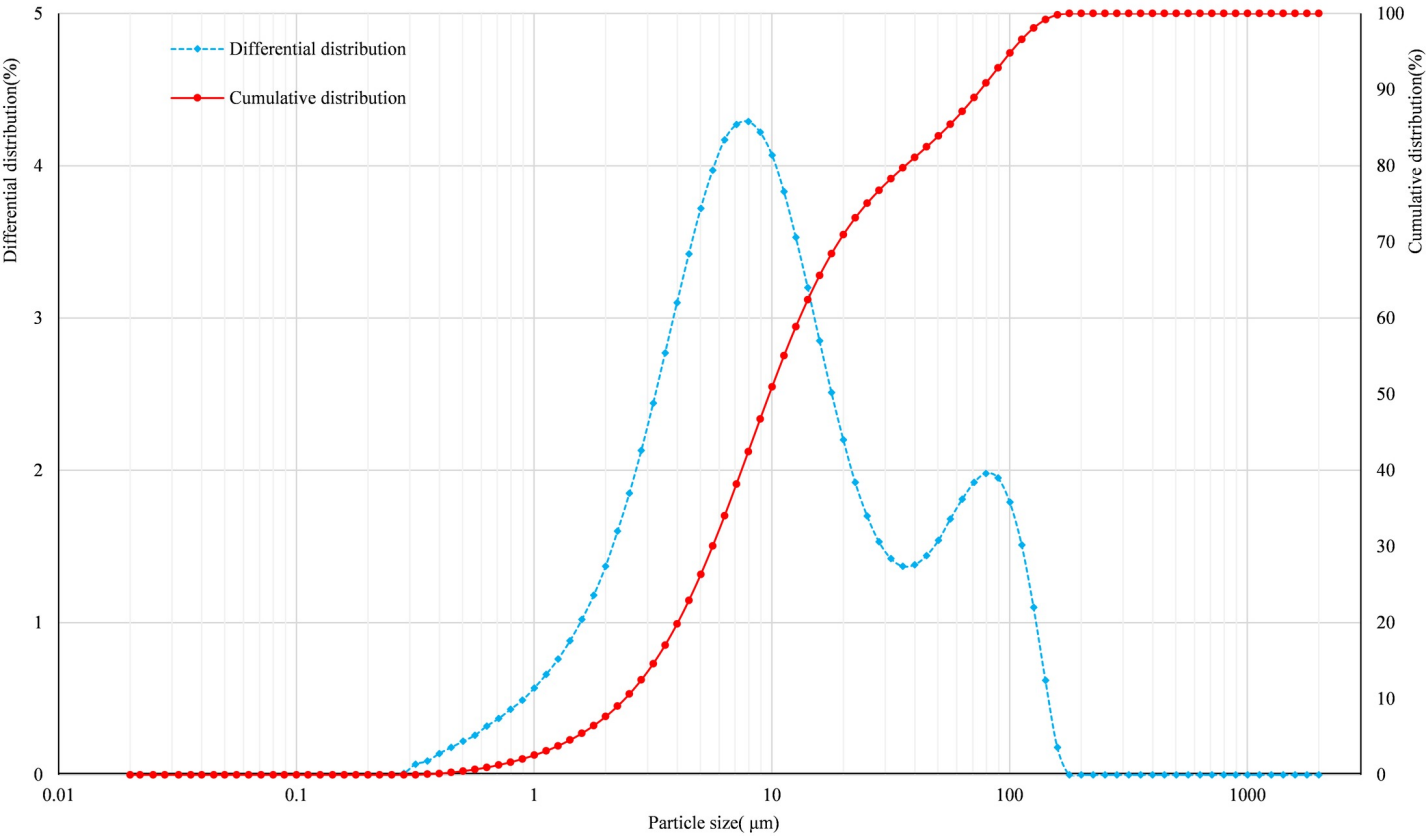
Particle size distributions of the unclassified tailings.

Generally, the physicochemical properties of backfilling aggregate have an important influence on the backfilling. A laser granulometer (Winner 2308A) was used to measure the particle size of the unclassified tailings. The chemical composition was measured by the X-ray diffraction (XRD) spectrometer (SIMENS D500); the specific gravity was measured by the pycnometer; the dry density was determined by electron densitometer (ET-03L); the permeability coefficient was determined by a permeability meter (TST-70) [23]. Fig 1 describes the particle size distribution of the unclassified tailings, and the primary physical properties and the chemical composition are listed in Table 1 and Table 2, respectively. Almost all of the tailings particles are less than 0.1 mm, and approximately 70.96 wt.% of the particle size is less than 0.020 mm. Hence, the tailings could be classified as ultrafine tailings, which would cause tremendous difficulties during settling. Because of the high content of fine mud in the unclassified tailings, the permeability coefficient is small, which is not conducive to the settling of unclassified tailings. The nonuniform coefficient is 5.549, i.e., the particle size is very uniform.

**Table 1:** Physical properties of the unclassified tailings.

Specific gravity	Dry density (g/cm^3^)	Permeability coefficient (cm/s)	Median size (μm)	Nonuniform coefficient	Curvature coefficient
2.85	1.65	3.86×10^−6^	9.759	5.549	0.983537

Nonuniform coefficient: Cc=d30*d30d60*d10; Curvature coefficient: Cc=d60d10. d_10_ (d_30_, d_60_): The mass of particles that particle size is smaller than d10 (d_30_, d_60_) is 10% (30%, 60%) of the total mass of the particles on the particle size distributions curve.

**Table 2:** Chemical composition of the unclassified tailings (unit in wt.%).

SiO_2_	CaO	Al_2_O_3_	MgO	Fe_2_O_3_	others
54.67	13.02	29.11	0.74	3.44	4.02

(2) Flocculants

The unclassified tailings slurry was weakly alkaline. According to the mechanism of "bridging attraction" [24–29], the flocculation of anionic flocculants is obviously higher than that of non-ionic flocculants and cationic flocculants in weakly alkaline solution [30, 31]. Therefore, the flocculants selected in this study are anionic polyacrylamide (APAM) with molecular weights of 5 million (A-050, Suzhou, China), 8 million (A-080, Suzhou, China), and 12 million (A-120, Suzhou, China) and poly-aluminum chloride (PAC, Suzhou, China).

#### 2.1.2. Flocculant solution preparation

2g APAM or PAC was dissolved into 1000ml water, and stirred with a stirrer at 40 r/min for 60 min to get flocculant solution with a concentration of 0.2 wt.%. The flocculant solution continued to be diluted to the desired concentration.

### 2.2. Test methods

#### 2.2.1. Flocculant selection experiments

The flocculant type has a great influence on the settling behavior of the unclassified tailings [32]. It is important to select the flocculant type that is appropriate for the unclassified tailings through experiments. Five sets of experiments were designed: no flocculants; APAM with a molecular weight of 5 million, 8 million, and 12 million added at a rate of 20 g/t; and PAC added at a rate of 40 g/t. The slurry concentration of the unclassified tailings in the experiments was 6.0%. Firstly, tailings and water were added to 1000 ml measuring cylinder and mixed for 1 min to obtain the slurry with a concentration of 6.0 vol.%. Then, the flocculant solution was added with a pipette and stirred for 20 seconds. The solid-liquid interface and the supernatant were observed. The flocculant type was qualitatively determined by the settling velocity of the interface and the supernatant clarity, without calculating the specific settling velocity. The selected flocculant would be used for the static cylinder experiments and the dynamic experiments.

#### 2.2.2. Static cylinder experiments

To study the effect of slurry concentration, flocculant unit consumption (the weight of flocculant added per ton of tailings) and flocculant concentration on the flocculating settling of the unclassified tailings, an orthogonal experiment was designed. The orthogonal experiment is a method that studies the influences of multiple factors and multiple levels [33]. Using the principles of the orthogonal experiment, the orthogonal table of L16 (4^3^) was chosen to implement the static cylinder experiments. The three factors are (A) slurry concentration, (B) flocculant unit consumption, and (C) flocculant concentration. Every factor has four levels, and they are as follows: 5 vol.% (A1), 6 vol.% (A2), 7 vol.% (A3), and 8 vol.% (A4); 2 (B1), 4 (B2), 6 (B3), and 8 (B4); and 0.06 wt.% (C1), 0.10 wt.% (C2), 0.14 wt.% (C3), and 0.18 wt.% (C4). Firstly, tailings and water were added to 1000ml measuring cylinder in a specific proportion and mixed for 1 min to obtain slurries. Then, the flocculant solution was added to the slurry with a pipette and stirred for 20 seconds. After the stirring was stopped, the height of the solid-liquid interface at different time was recorded. In order to eliminate the influence of mud height, the unclassified tailings mass weighed should be the same when preparing the unclassified tailings slurry with different concentrations.

#### 2.2.3. Dynamic experiments

**Fig 2:**
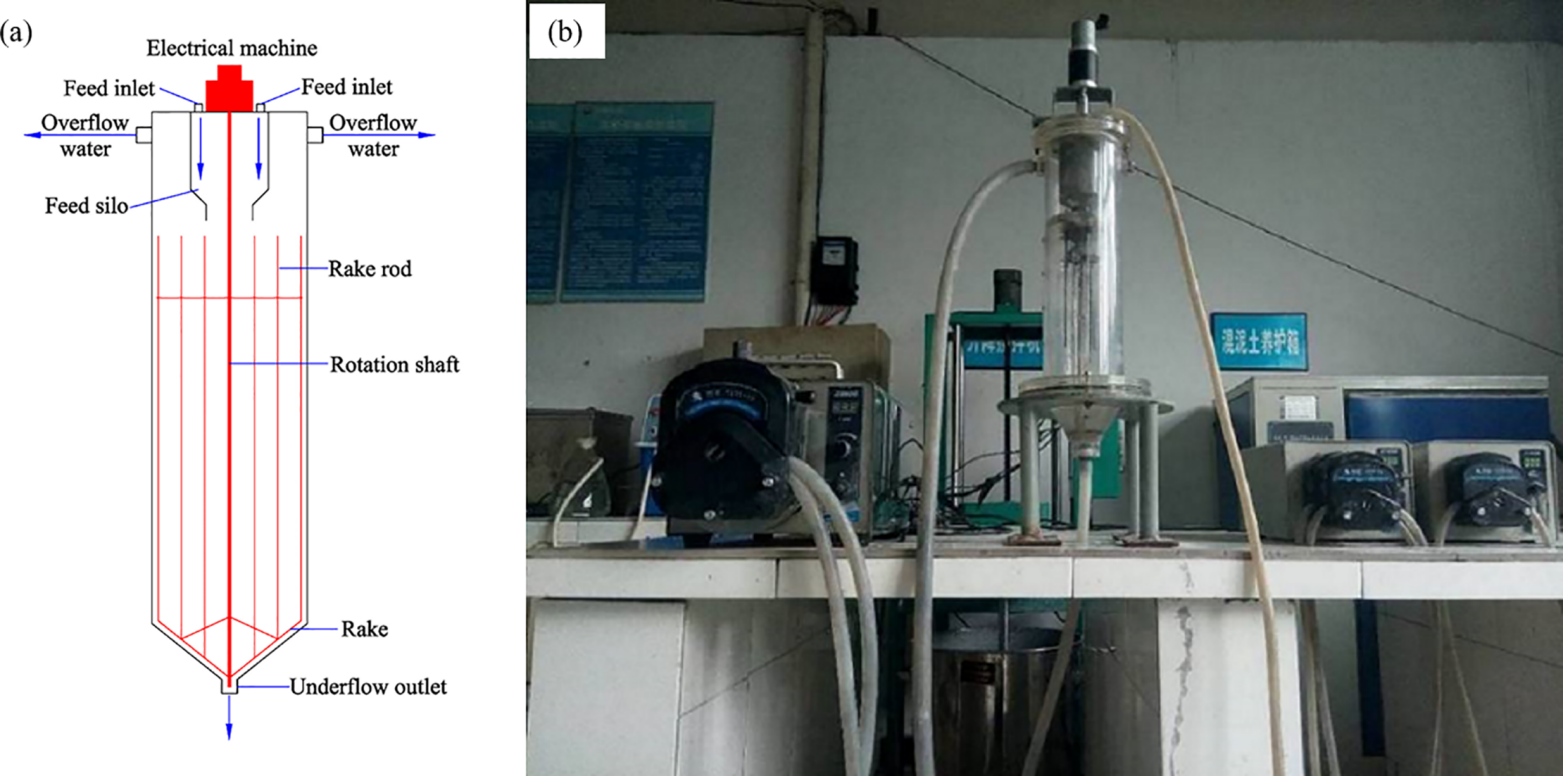
Dynamic experimental apparatus. (a) Schematic diagram; (b) Working photograph.

To study the effect of feed velocity, mud height and rake speed on the flocculating settling of the unclassified tailings, dynamic experiments are required. The dynamic experimental apparatus consists of a thickener, three peristaltic pumps and several tubes. The thickener has a 100 mm diameter, the upper part has a feed inlet, an overflow port is connected to the sidewall, the front of the barrel wall is graduated, and an inner rake with adjustable speed is provided. Three peristaltic pumps are used to pump the unclassified tailings slurry and flocculant solution into the feed silo of the thickener, and pump out the underflow samples from the bottom of the experimental apparatus; this apparatus achieves continuous feeding and continuous discharging. The dynamic experimental apparatus is shown in Fig 2. The tailings and water were placed in a mixing tank and continuously stirred to prevent segregation. After the experiment started, the tailings slurry and the flocculant solution were continuously pumped into the thickener at the set speed and flocculant unit consumption by controlling the peristaltic pump, and then the rake speed was adjusted. When the mud height reached the set height, the underflow was pumped out from the bottom of the thickener, and the overflow water was sampled. The appropriate amount of overflow water was dried to constant weight to obtain the suspended solids concentration of the overflow water; the appropriate amount of the underflow was dried to constant weight to obtain the underflow concentration.

According to the result of static cylinder experiments, the slurry concentration, flocculant concentration and flocculant unit consumption were set. Considering the experiences of previous related research and industrial practice [34, 35], feeding speed was set to 0.2 L/min, the rake speed was set to 0.2 r/min, and the mud height was set to 120 mm. The control variate method was applied to the dynamic experiments. In other words, when the mud height and rake speed are set value, the underflow concentration and the suspended solids concentration with different feed velocity are measured; when the feed velocity and rake speed are set value, the underflow concentration and the suspended solids concentration with different mud height are measured; when the mud height and feed velocity are set value, the underflow concentration and the suspended solids concentration with different rake speed are measured. The parameters were adjusted to the optimal value based on the experimental results. The evaluation indices of settling behavior are the underflow concentration and the suspended solids concentration in the overflow water.

## 3. Results and discussion

### 3.1. Analysis of the flocculant selection experiments

**Fig 3:**
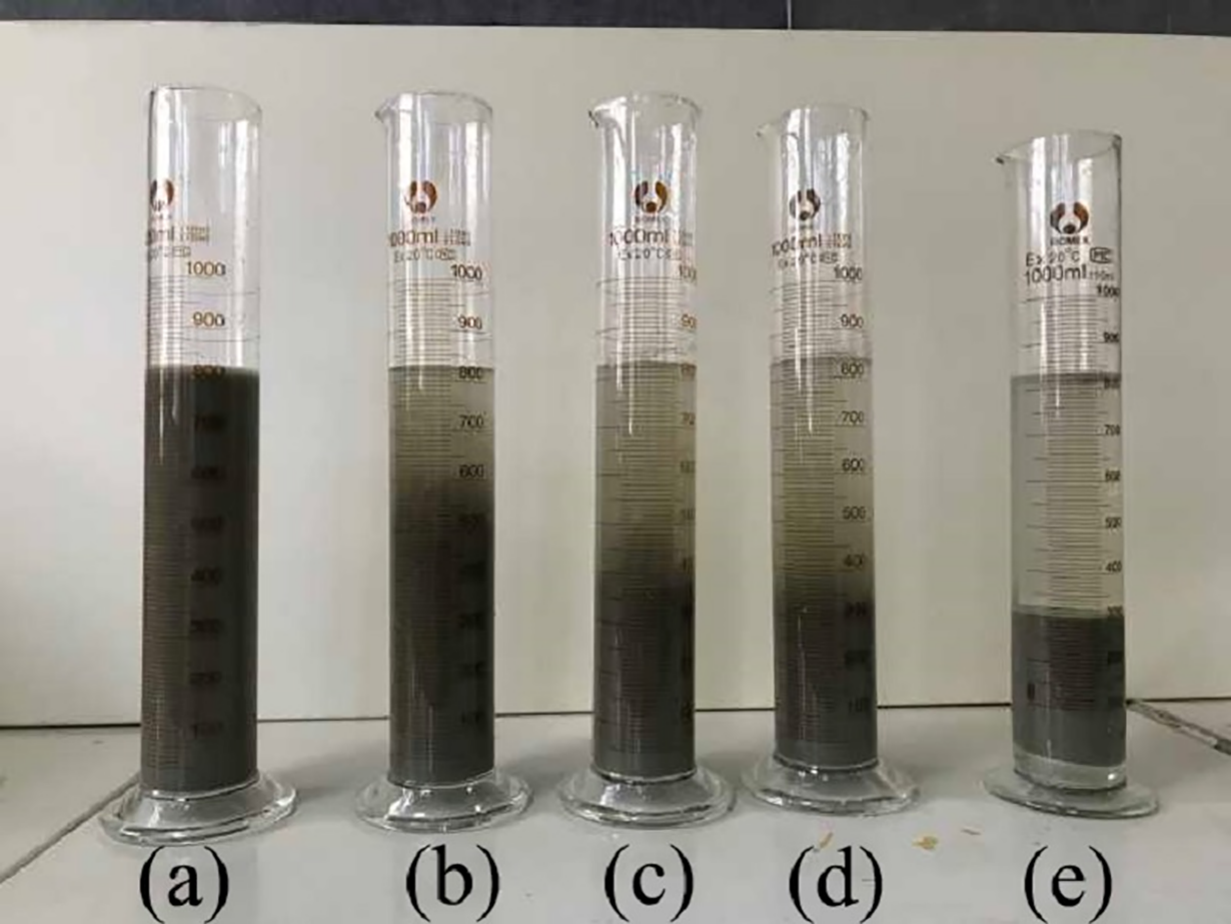
Settling behavior with different floculants. (a) no flocculants; (b) PAC; (c) APAM with a molecular weight of 5 million; (d) APAM with a molecular weight of 8 million; (e) APAM with a molecular weight of 12 million.

**Fig 4:**
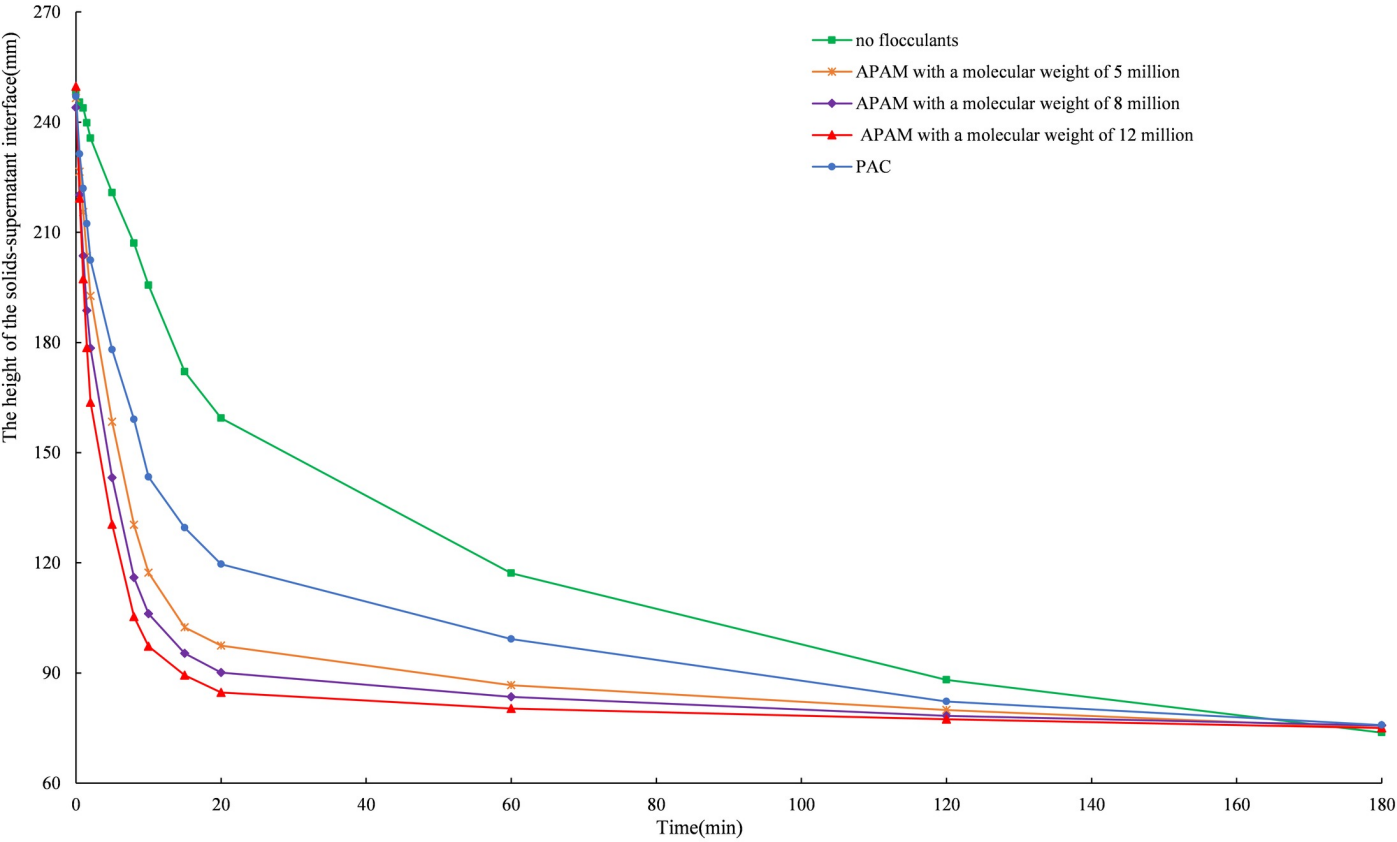
Height curves of the solid-liquid interface.

There is a clear solid-liquid interface between the supernatant and the tailings particles during settling, and the settling process is the process of the settlement of this interface [36]. The results of flocculant selection experiments are shown in Fig 3, and the settling curve is shown in Fig 4. It can be found that all four flocculants can improve the settling effect of the unclassified tailings slurry. APAM is better than PAC in terms of settling velocity and supernatant clarity, i.e., APAM has a significant effect on the flocculating settling of the unclassified tailings. Therefore, APAM with a molecular weight of 12 million is recommended as the flocculant for the settling of the unclassified tailings in the mine.

### 3.2. Analysis of static cylinder experiments

The settling is an unsteady process. The solids-supernatant interface rapidly decreased within 1 min after adding APAM, and then the settling velocity slows down, and the solid-liquid interface changed little after 20 min. Therefore, the maximum velocity was used to characterize the velocity characteristics of the flocculating settling, and the limit mass concentration was used to characterize the concentration characteristics of the flocculating settling. The evaluation indices are the settling velocity and the underflow concentration. The settling velocity in the first 1min was the highest; the velocity at this time was chosen as the settling velocity. The settling velocity is the settling distance of the solid-liquid interface per unit time. The underflow concentration did not change after three hours; the concentration at this time was chosen as the limit mass concentration. The settling velocity and the underflow concentration of each group are summarized in Table 3. The variance of the settling velocity and the underflow concentration was analyzed by Statistical Product and Service Solutions (SPSS) software, and the analysis results are listed in Table 4. When the significance level is greater than 0.05, the influence of the factors is not significant.

**Table 3:** The results of the static cylinder experiments.

Case	Factors			Settling velocity (mm/s)	Underflow concentration (wt.%)
	Slurry concentration (vol.%)	Flocculant unit consumption (g/t)	Flocculant concentration (wt.%)		
	A	B	C		
1	5	10	0.06	8.481	63.144
2	5	20	0.1	9.168	62.446
3	5	30	0.14	9.564	61.512
4	5	40	0.18	9.107	59.015
5	6	10	0.1	8.205	62.292
6	6	20	0.06	8.358	61.814
7	6	30	0.18	9.027	61.159
8	6	40	0.14	8.741	58.484
9	7	10	0.14	7.279	63.048
10	7	20	0.18	7.572	61.653
11	7	30	0.06	7.675	61.957
12	7	40	0.1	7.229	58.761
13	8	10	0.18	5.714	62.746
14	8	20	0.14	5.803	61.768
15	8	30	0.1	6.487	61.355
16	8	40	0.06	6.032	59.079

**Table 4:** Variance analysis of the static cylinder experiments.

Factor	Dependent Variable	Sum of Class III Squares	Freedom	Mean Square Deviation	F	Significance
A	Settling velocity	22.349	3	7.450	225.596	0.000
	Underflow concentration	0.744	3	0.248	4.353	0.060
B	Settling velocity	1.198	3	0.399	12.090	0.006
	Underflow concentration	35.072	3	11.691	205.082	0.000
C	Settling velocity	0.123	3	0.041	1.240	0.375
	Underflow concentration	0.303	3	0.101	1.774	0.252

#### 3.2.1. Analysis of the settling velocity

Table 3 shows that factor C has no significant effect on flocculating settling velocity of the unclassified tailings, and the significance order is A>B>C. In other words, the effect of the slurry concentration on flocculating settling velocity of the unclassified tailings is the largest, followed by the flocculant unit consumption and then the influence of the flocculant concentration.

**Fig 5:**
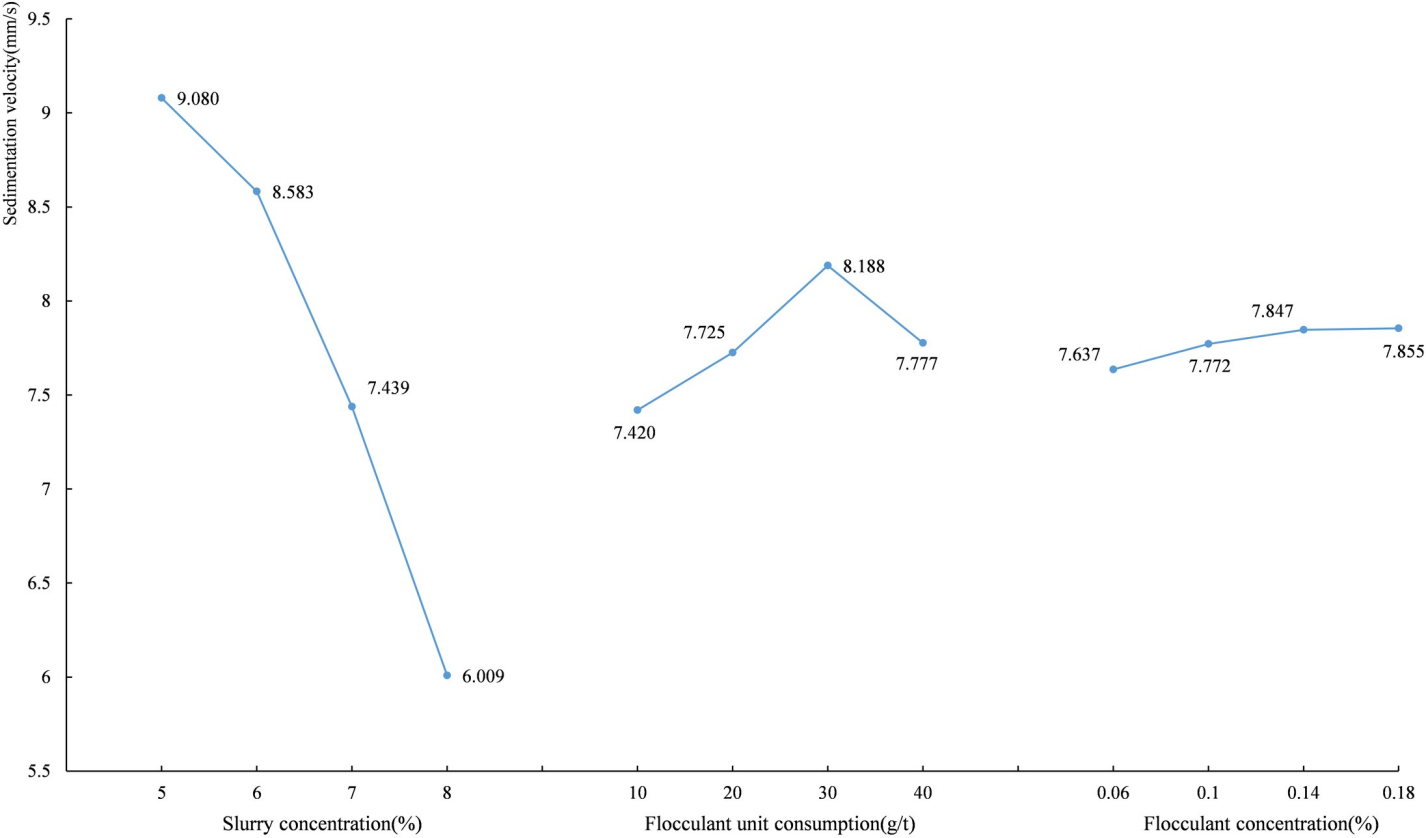
The average settling velocity of the unclassified tailings at each level.

The average settling velocity (Taking A1 as an example, the average settling velocity is the average of all settling velocities corresponding to a slurry concentration of 5 vol.%, regardless of B and C.) value corresponding to each level are shown in Fig 5. Fig 5 shows that the flocculating settling velocity of unclassified tailings decreases with the increase of slurry concentration and that the flocculating settling velocity increases first and then decreases with the increase of flocculant unit consumption. The tailings settling is in hindered settling [17]. The greater the tailings slurry concentration is, the more serious the hindered settling of the tailings particles is, and the greater the chance of collision with each other is; thus, a high tailings slurry concentration greatly slows down the settling velocity. The flocculant acts as a bridge by electrically neutralizing the surface charge of the tailings particles and reduces the repulsive force between the tailings particles by compressing the double layer of particles, which forms the flocs between the tailings particles and the flocculating and the tailings particles [29, 30], thereby accelerating the settling velocity. When the flocculant unit consumption is low, some tailings particles cannot interact with the flocculants, resulting in slower settling. As the flocculant unit consumption increases, the tailings particles and the flocculants can become fully integrated, thereby increasing the settling velocity. When the flocculant unit consumption is too high, the viscosity of the tailings slurry further increases, which causes both the flocs settling resistance and the hindered settling to increase, resulting in the reduction of settling velocity.

#### 3.2.2. Analysis of the underflow concentration

Table 3 shows that factor A and factor C have no significant effect on flocculating settling velocity of the unclassified tailings, and the significance order is A>B>C. In other words, the effect of slurry concentration on the underflow concentration is the largest, followed by the flocculant unit consumption and then the influence of the flocculant concentration.

**Fig 6:**
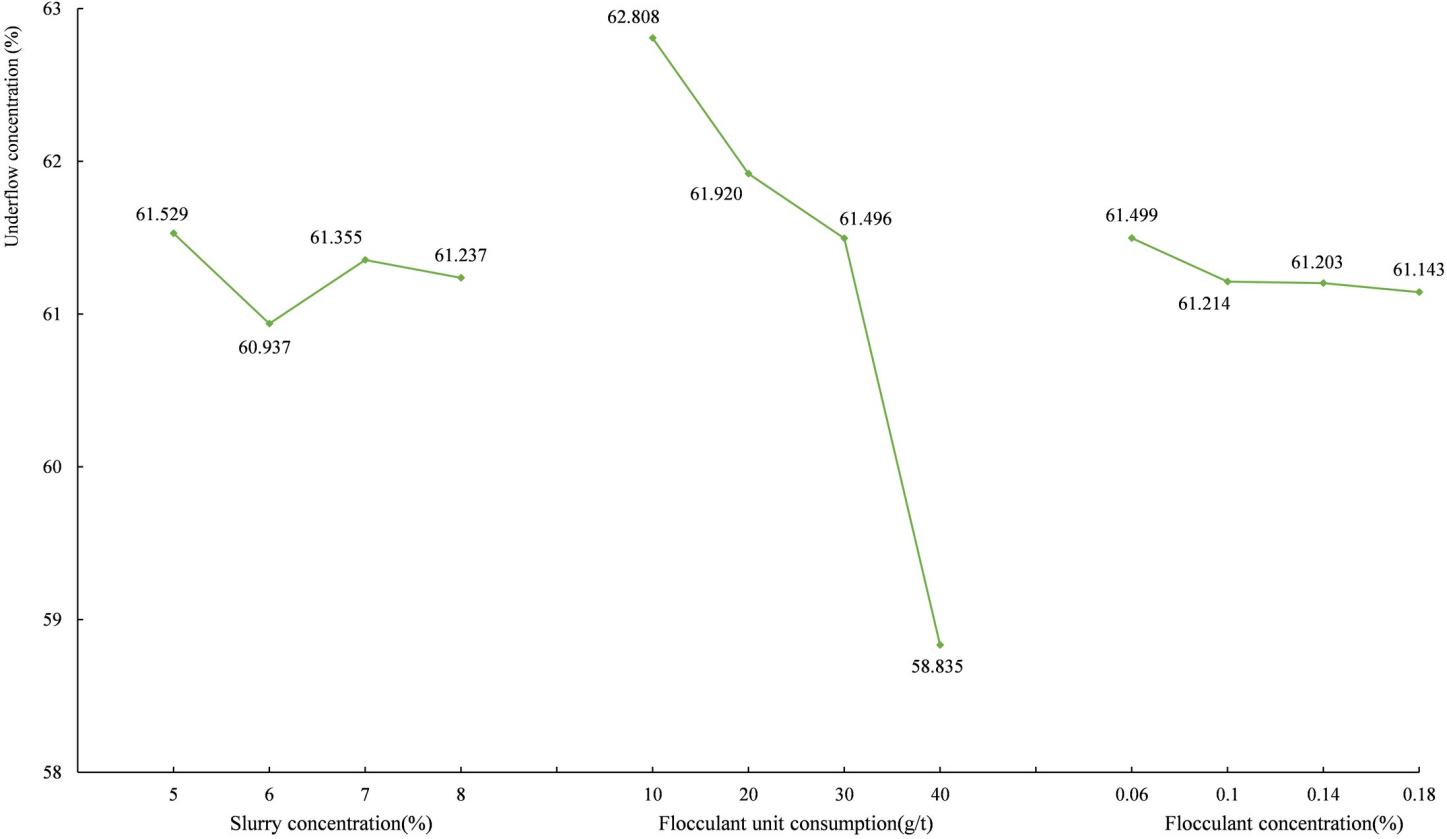
The average underflow concentration of the unclassified tailings at each level.

The average underflow concentration values corresponding to each level are shown in Fig 6. Fig 6 shows the underflow concentration is positively correlated with the slurry concentration and negatively correlated with the flocculant unit consumption and flocculant concentration. When the flocculant is excessive, the surface of the particles is completely occupied by the flocculant molecules, and the particles repel each other due to the steric hindrance effect of the polymer adsorption film [37]. And much trapped water exists inside the flocs or between the flocs [30, 31]. It is difficult to break the static balance between water and particles and to release the trapped water, resulting in the reduction of the underflow concentration.

#### 3.2.3. Parameters analysis

**Fig 7:**
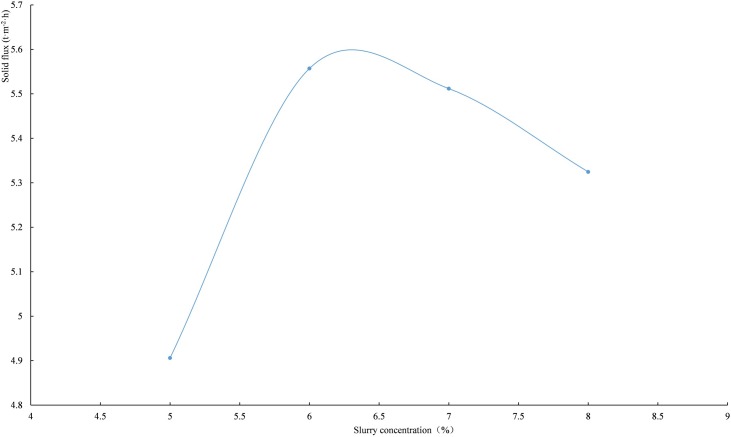
The relationship between solid flux and slurry concentration.

It is inaccurate to determine the slurry concentration only using settling velocity because the settling velocity cannot reflect the processing capacity per unit area of the settling apparatus [38]. The solid flux [39] is the mass of solids that pass through the unit cross-sectional area in unit time and is related to slurry concentration and settling velocity. The calculation equation is as follows:

$$G=\frac{m}{St}=\frac{3.6\varphi_{v}\rho_{S}vSt}{St}=3.6\rho_{S}\varphi_{v}v$$ (1)

where *G* is the solid flux (t/(m^2^·h)), *S* is the cross-section (m^2^), *t* is the time (h), *m* is the solid mass (t), *ρ*_*s*_ the density of the unclassified tailings (g/cm^3^), *v* is the settling velocity (mm/s), and *φ*_*v*_ is the volume concentration of the slurry. The lower the slurry concentration is, the faster the settling velocity is; thus a maximum of solid flux can be found. When the flocculant unit consumption is 30 g/t, the solid flux corresponding to different slurry concentration is calculated. As shown in Fig 7, there is an optimal value for the solid flux between 6 vol.% and 7 vol.% of slurry concentration, with an optimal at approximately 6 vol.%.

Considering the settling velocity and the underflow concentration, the flocculant unit consumption is 30g/t. When the flocculant concentration is 0.06 wt.% ~ 0.18 wt.%, the flocculating settling effect is less affected; thus, the flocculant concentration is determined to be 0.1 wt.%.

In summary, the optimal parameters obtained by the static cylinder experiments are as follows: the flocculant unit consumption is 30 g/t, the slurry concentration is 6 vol.%, and the flocculant concentration is 0.1 wt.%.

### 3.3 Analysis of the dynamic experiments

The evaluation indices used in dynamic experiments are the underflow concentration and the suspended solids concentration in the overflow water. *Integrated wastewater discharge standard* [40] requires the suspended solids concentration of wastewater in mining, mineral processing, and coal preparation industrial discharge cannot be higher than 300 mg/L.

#### 3.3.1. Feed velocity

**Fig 8:**
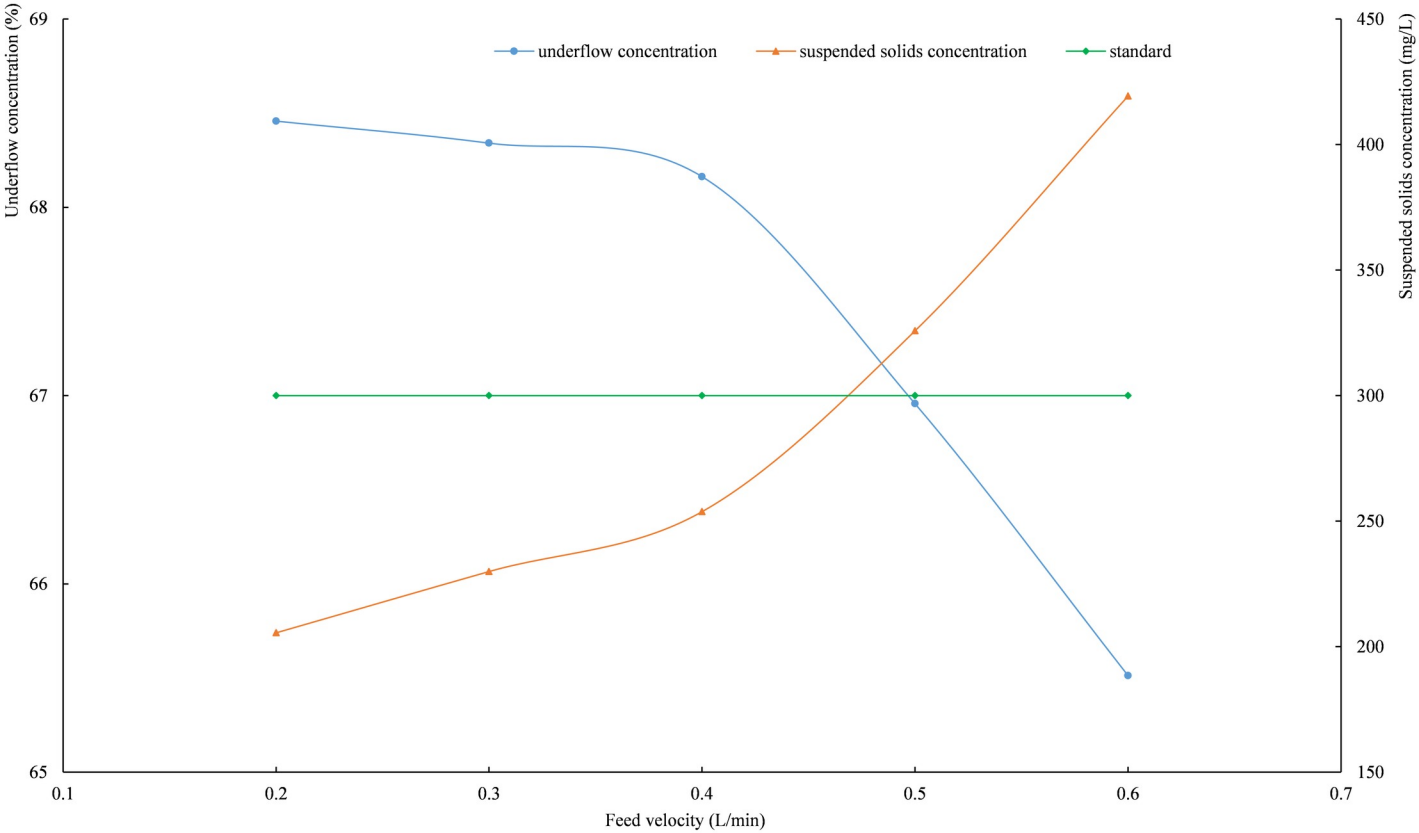
The relationships among underflow concentration, suspended solids concentration and feed velocity. (*Integrated wastewater discharge standard* requires the suspended solids concentration of wastewater in mining, mineral processing, and coal preparation industrial discharge cannot be higher than 300 mg/L.).

When the rake speed is 0.2 r/min and the mud height is 120 mm, the curve of the settling indices are shown in Fig 8 with the feed velocity changes. As shown in Fig 8, slower feed velocity could produce higher concentration underflow but lower clarity overflow water. When the feed velocity is greater than 0.47 L/min, the suspended solids concentration in the overflow water is higher than 300 mg/L.

When the feeding velocity is low, the tailings slurry and flocculant solution remain in the experimental apparatus for a long time, and the tailings particles and flocculants become fully integrated, thereby improving the effect of flocculating settling. With the increase in feed velocity, the content of tailings particles gradually increases. Limited by the capacity of the experimental apparatus, part of tailings particles cannot be attracted by the flocculants and flow out with the overflow water; as a result, the suspended solids concentration in the overflow water increases and the underflow concentration decreases.

#### 3.3.2. Mud height

**Fig 9:**
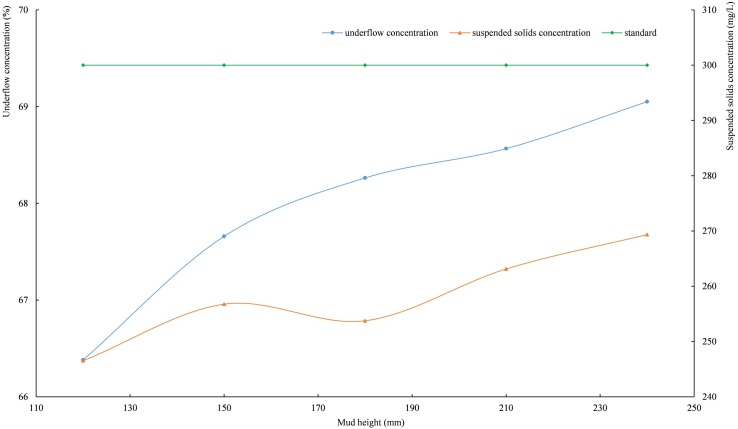
The relationships among underflow concentration, suspended solids concentration and mud height. (*Integrated wastewater discharge standard* requires the suspended solids concentration of wastewater in mining, mineral processing, and coal preparation industrial discharge cannot be higher than 300 mg/L.).

When the rake speed is 0.2 r/min and the feed velocity is 0.4 L/min, the curves of the settling indices are shown in Fig 9 with the mud height changes. As shown in Fig 9, the greater the mud height, the higher the underflow concentration and the suspended solids concentration in the overflow water; and the suspended solids concentration in the overflow water is always less than 300 mg/L.

The tailings particles in the underflow are loosely arranged [41], and a large amount of pore water exists between the particles. Under the gravity action of the upper particles, part of the pore water can be slowly squeezed out and released to the mud top. The increase in the mud height is followed by an increase in the mud pressure [42, 43]. The pore water between particles is more easily released, and the underflow concentration further increases. For specific equipment, the mud height determines the supernatant height, i.e., the higher the mud height is, the shorter the supernatant height is. However, the supernatant height has a direct relationship with the flocculating settling; thus, the suspended solids concentration in the overflow water slowly increases with the increase in the mud height.

#### 3.3.3. Rake speed

**Fig 10:**
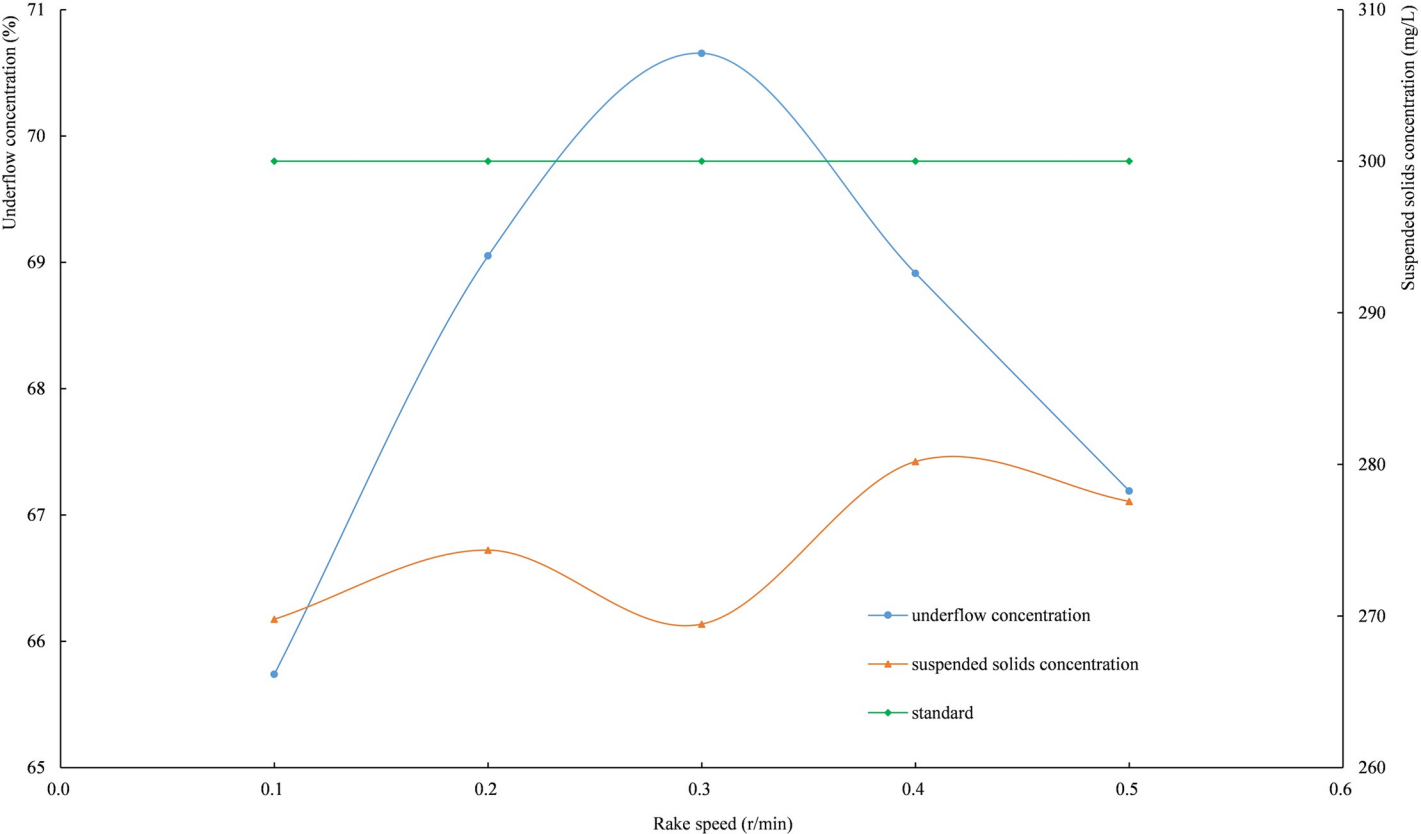
The relationships among underflow concentration, suspended solids concentration and rake speed. (*Integrated wastewater discharge standard* requires the suspended solids concentration of wastewater in mining, mineral processing, and coal preparation industrial discharge cannot be higher than 300 mg/L.).

When the feed velocity is 0.4 L/min and the mud height is 240 mm, the curve of the settling indices shown in Fig 10 changes with the rake speed. As shown in Fig 10, the underflow concentration has a maximum at the rake speed of 0.3 r/min, and the rake speed has little effect on the suspended solids concentration in the overflow water.

The rotation of the rake can shear the underflow [44–46], and the effective disturbance can break the force balance of flocs or particles. The trapped water between tailings particles and flocs or among flocs can be expelled upward along with the pore water. At the same time, the loose tailings particles are rearranged, and the fine particles fall into the pores, causing the underflow concentration to increase. The rotating rake rod forms some water conductors in the passing mud layer. When the rake speed is low, the disturbance is not sufficient to completely release the trapped water. As a result, the water conductor is blocked again before the pore water reaches the mud top, and the water-conducting effect is inhibited, resulting in a lower concentration of the underflow. When the rake speed is too high, the excessive disturbance of the rake on tailings or flocs hinders the tailings particles sinking further and weakens the compaction effect of the underflow; thus, the underflow concentration decreases.

#### 3.3.4. Parameters analysis

According to the feed velocity, the equation of solid flux is derived. The solid flux corresponding to the feed velocity is calculated; this solid flux value can provide the basis for the calculation of feed velocity of the subsequent engineering applications.

$$G=\frac{m}{St}=\frac{0.06\rho_{S}\varphi_{v}Qt}{St}=0.06\rho_{S}\varphi_{v}\frac{Q}{S}$$ (2)

where *Q* is the feed velocity (L/min). As can be seen from Eq (2), the larger the diameter of the dynamic settling apparatus is, the greater the processing capacity is, and the greater the required feed velocity is. The feed velocity required for the industrial tests can be calculated using the solids flux obtained by the dynamic experiments. The solid flux can effectively reflect the feed velocity required for different cross-section. According to the experimental results, to ensure the effect of dynamic settling flocculation, the feed velocity cannot be greater than 0.4 L/min; In other words, the solid flux cannot be greater than 0.523 t/(m^2^·h).

With the increase of mud height, the underflow concentration of dynamic flocculating settling gradually increases; however, the mud height cannot infinitely increase because of the limitations of experimental apparatus. The underflow concentration cannot be less than 70 wt.% according to the recommended proportioning parameters that are determined by the strength requirements of the backfilling body and the fluidity requirements of the backfilling slurry. When the rake speed is 0.3 r/min, the feed velocity is 0.4 L/min and the mud height is 240 mm, the underflow concentration reaches 70 wt.%. The mud height should be higher than 2 m in practical applications, and the underflow concentration can reach more than 70 wt.% after thickening.

The rake speed should be controlled to approximately 0.3 r/min, the feed velocity cannot be greater than 0.4 L/min and its solid flux cannot be greater than 0.523 t/(m^2^·h), according to the analysis of the underflow concentration and the suspended solids concentration of in the overflow water.

## 4. Industrial tests

The slurry concentration, flocculant unit consumption and flocculant concentration on the flocculating settling of unclassified tailings were obtained by static cylinder experiments; the effects of feed velocity, mud height and rake speed on the flocculating settling of unclassified tailings were analyzed by using the dynamic experimental apparatus of thickener. It is necessary to verify the reliability of the above experimental results by industrial tests before engineering applications.

**Fig 11:**
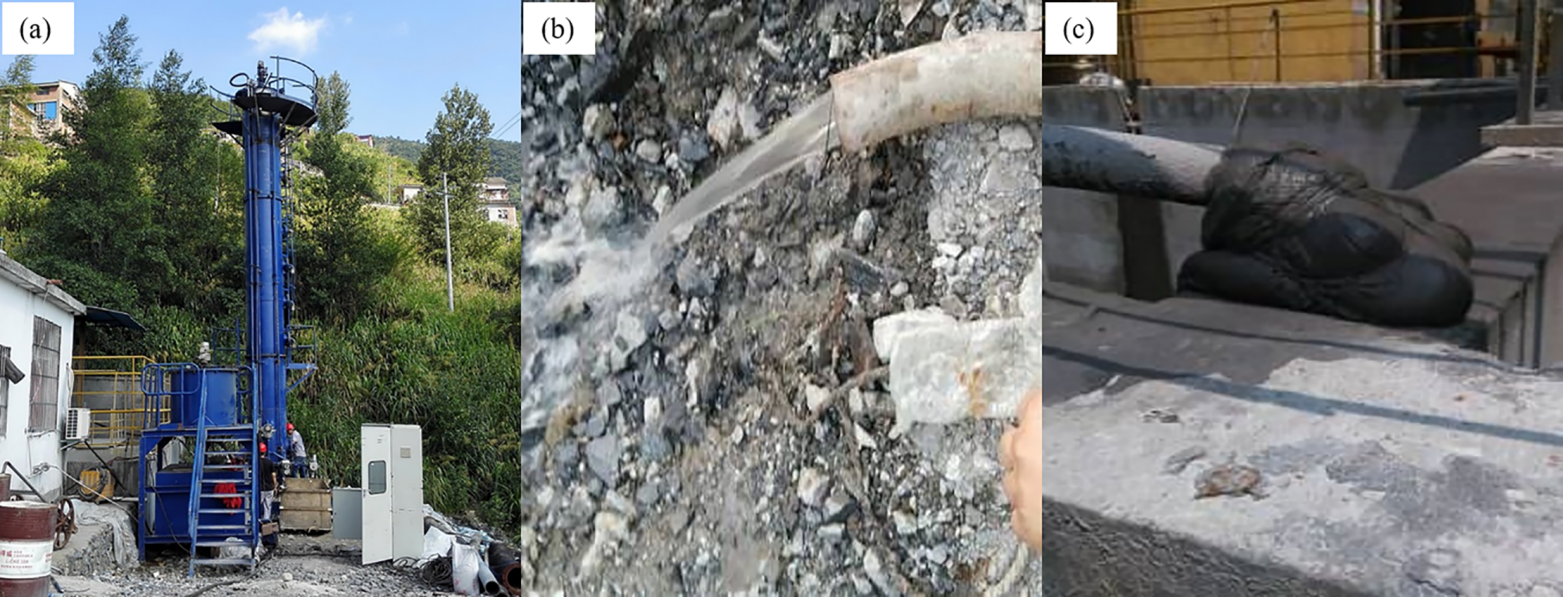
The process of industrial tests. (a) Deep cone thickener; (b) Overflow water; (c) Underflow.

**Fig 12:**
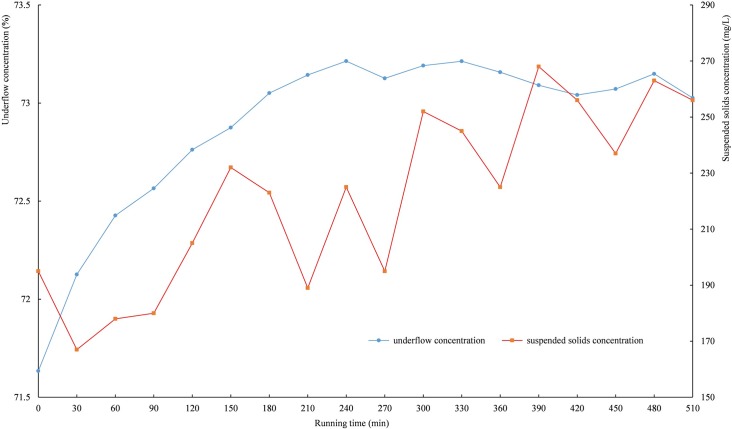
The Curve of the underflow concentration and suspended solids concentration in the overflow water with the running time.

The deep cone thickener with the diameter of 1 m was used for industrial tests. The slurry used in industrial tests was supplied by the beneficiation plant of Yinshan Copper Mine. Because the slurry concentration is approximately 10 vol.%, recycled water is used to dilute the slurry, and the diluted slurry is pumped into the deep cone thickener. The parameters of the flocculating settling decided by static cylinder experiments and dynamic experiments are as follows: the flocculant type is APAM with a molecular weight of 12 million, the flocculant unit consumption is 30 g/t, the slurry concentration is 6 vol.%, the flocculant concentration is 0.1 wt.%, the rake speed is 0.3 r/min, and the feed velocity is 40 L/min. In practical applications, when the underflow is discharged at a higher speed and the mud height is low, the mud is easily penetrated; thus, the mud height cannot be less than 2 m under normal circumstances. During the tests, after the mud height reached 2 m, the underflow concentration and the suspended solids concentration in the overflow water were measured every 30 minutes. The mud height was controlled at 4 m by adjusting the velocity at which the underflow is discharged. The process of industrial tests is shown in Fig 11, and the underflow concentration and suspended solids concentration in the overflow water with the running time are shown in Fig 12. The underflow concentration ranges from 71.8 wt.% to 73.3 wt.%, and the suspended solids concentration ranges from 200 mg/L to 270 mg/L, which meets the requirements of industrial applications.

## 5. Conclusions

In this work, the effect of slurry concentration, flocculant unit consumption and flocculant concentration, feed velocity, mud height and rake speed on the flocculating settling of unclassified tailings were systematically studied through the static cylinder experiments and the dynamic experiments. The dynamic experimental apparatus was used to continuous feeding and continuous discharging, which is closer to practical application. The settling behaviors were evaluated by four indexes: underflow concentration, sedimentation velocity, solid flux and overflow water solid content. It is more reasonable to evaluate the settling behaviors of unclassified tailings by four indexes: settling velocity, underflow concentration, solid flux and suspended solids concentration in the overflow water. From the study results, the following conclusions can be drawn:

1. The static cylinder experiments show that the flocculating settling velocity of unclassified tailings decreases with the increase of slurry concentration and that this velocity increases first and then decreases with the increase of flocculant unit consumption. The underflow concentration is positively correlated with the slurry concentration and negatively correlated with the flocculant unit consumption and flocculant concentration.
2. The dynamic experiments show that slower feed velocity could produce higher concentration underflow but lower clarity overflow water. The greater the mud height, the higher the underflow concentration and the suspended solids concentration in the overflow water. The underflow concentration has a maximum at the rake speed of 0.3 r/min, and the rake speed has little effect on the suspended solids concentration in the overflow water.
3. The parameters of flocculating settling determined by static cylinder experiments and dynamic experiments are as follows: the flocculant type is APAM with a molecular weight of 12 million, the flocculant unit consumption is 30 g/t, the slurry concentration is 6 vol.%, the flocculant concentration is 0.1 wt.%, the rake speed is 0.3 r/min, and the feed velocity is 0.4 L/min (its solid flux is 0.523 t/(m^2^·h)). The industrial tests verified the reliability of the experimental results.
4. The optimal flocculation sedimentation parameters can be determined by the study, which not only provides theoretical basis for the selection of deep cone thickener, but also has important guiding significance for the stable and reliable operation of backfilling system.

## Supporting information

S1 File Data. (XLSX)
